# Supplementary material for: Patterns of Intron Gain and Loss in Fungi
Source: PLoS Biol. 2004 Nov 30;2(12):e422. doi: 10.1371/journal.pbio.0020422 (PMC532390; doi:10.1371/journal.pbio.0020422)
Supplement: Table S1 — Also available at http://genes.mit.edu/NielsenEtAl/. (4.3 MB ZIP). [file pbio.0020422.st001.zip › NielsenEtAl/html/328.html]

AN6162.1.NCU00034.1.MG05331.1.FG06689.1


```
 CLUSTAL W (1.82) Multiple Sequence Alignments - Introns Inserted


Sequence 1: NCU00034.1	313 aa
Sequence 2: MG05331.1	341 aa
Sequence 3: FG06689.1	282 aa
Sequence 4: AN6162.1	304 aa
Alignment Length: 350 aa
Number Identitical Residues: 88 aa
Alignment Score (without introns) 5151


MG05331.1 	MPACVVDAAAVNGRGHGGRDISWADGSGVECSATCSSAPLAQPTRRRRSRQFFVPPPPSP
NCU00034.1	---------------------------MSETPSSSSSAPLATGRSDGPSVHHRQHSRGQT
FG06689.1 	------------------------------------------------MAETFQPAGARP
AN6162.1  	----------------------------MANDTAVPSASSATPT--AAAYNTWTPPNPSL
          	                                :: .::. :         .    .    

MG05331.1 	INVNGAGPAHTTGPKNHEAMTKTDLTLPVLSPIAQSASAANSPVHPYMPGQPKSLAGIAM
NCU00034.1	THIN-IPAATATATATATHLSEADLSLPTDSPTSANSDLNLVNDRDYFPYQPKSLAGIAT
FG06689.1 	VNFSPSAAHHQAALSHHDSFPGNSRSLD-----------ELMILKPYFAGQPKSLSGIAM
AN6162.1  	SQSQLQPQSQSQSQSQPIPITNKTTSTD----------------ALLYPSGSKSLSGISL
          	 : .        .      :.    :                      .  .***:**: 

MG05331.1 	RAFCLGSVFAVSVVSTIPILLLTSSP-----LWRLPFFVGALATHHFLEFWTTAAYNTPA
NCU00034.1	RSFCLGIAVTIGVGATVATLFYTSSS-----LWRLPFFLAALATFHFLEFWTTAAYNTRA
FG06689.1 	RAFCLGIALASSGIAMTTILVMTSSP-----AWRVPFFLFSLSAFHFLEFWTTAEKNTLV
AN6162.1  	RAFLLGTTLGLSSALTISLLNLTISDSQPHYLWRLPFFLASLSLFHFLEYYITAAHNTRY
          	*:* ** ..  .     . *  * * :..   **:***: :*: .****:: **  **  

MG05331.1 	ANVDSYLLTANWPMQTIAYAFAALECLLVCLVSPSRAWAPLHTGP----LLLLLGLVLTA
NCU00034.1	ADVKSFLLTANWPGYAIAHGFATLECLLTNAFWPNRAWAPFGSGT----LLCLLGLALTV
FG06689.1 	ASIGSFLLTANWPGYAIAHSAAFLECAIVNIIFPERNWAPFGIGS----LLLVAGLLMVT
AN6162.1  	ADISAFLLSSNGWAYNVAHSSAALECLLGYVFFPHDSYFKFTASVGGVKVQAVLGLFLMI
          	*.: ::**::*     :*:. * *** :   . *   :  :  . .. .:  : ** :  

MG05331.1 	VGQLVRSAAMIQAGLSFNHIVQQARGPQHHLVTTGIYAKLRHPSYFGYFYWALGTQLVLG
NCU00034.1	IGQVVRSVAMVQAGPSFNHLVQRQRSSAHVLVTSGIYGRFRHPSYFGFFWWALGTQMAMG
FG06689.1 	VGQYVRSVAMLQAGASFNHHVQTRKKDSHELVTTGIYSIFRHPSYFGFFYWGLGTQLVMG
AN6162.1  	LGQTVRTLAMAQAGSNFNHTVQVERKEGHTLVQHGVYSVLRHPSYFGFFWWGLGTQLVLG
          	:** **: ** *** .*** **  :   * **  *:*. :*******:*:*.****:.:*

MG05331.1 	NLVSTIAYSFVLYRFFSRRIRH~EEETLVRFFGEEYNDYKRTVGTKIPFIP
NCU00034.1	NVVSFVAYAAVLWKFFSSRIKH~EEEFLVKFFGQEYVDYRRRVGTKIPFVP
FG06689.1 	NVLCFFAYAFVLWKFFNIRIKH~EEGKLIEFFKDDYVQYRKRVGTKIPFIA
AN6162.1  	NVVCFVGYAVVLWKFFNNRIQR1EERFLIAFFGNEYVEYKKRTIIGIPGIA
          	*::. ..*: **::**. **:: **  *: ** ::* :*:: .   ** :.
```
